# Practicality of Using Pressure Sensors and Accelerometers to Quantify Hand Orthosis Compliance at Home

**DOI:** 10.3390/bioengineering13060697

**Published:** 2026-06-18

**Authors:** Devi Baruni Devanand, Matthew D. Gardiner, Angela E. Kedgley

**Affiliations:** 1 Department of Bioengineering, Imperial College London, London SW7 2AZ, UK; 2 Kennedy Institute of Rheumatology, Nuffield Department of Orthopaedics, Rheumatology and Musculoskeletal Sciences (NDORMS), University of Oxford, Oxford OX3 7FY, UK; matthew.gardiner@kennedy.ox.ac.uk; 3 Department of Plastic Surgery, Wexham Park Hospital, Frimley Health NHS Foundation Trust, Slough SL2 4HL, UK

**Keywords:** orthosis, upper limb, compliance, adherence, pressure sensor, accelerometer, objective compliance monitoring, wear time, activities of daily living

## Abstract

Orthosis compliance monitoring provides insights into effective orthosis design and user wear time. Frequently, patient reports of orthosis use are subjective and often result in overestimation of compliance. Therefore, a tool to objectively observe whether patients wear their orthoses as instructed is vital. This study assessed the real-world practicality of using an objective compliance monitoring device with a hand orthosis. A device consisting of a pressure sensor and accelerometer was tested by ten healthy volunteers who wore a hand orthosis daily and completed a diary of their wear time and activities for a week. Sensor data obtained from the compliance monitoring device were analysed to discern each user’s orthosis wear time. Differences between estimated wear time and actual wear time were insignificant. Pressure-based wear time estimations had a specificity of 99.3 ± 0.7% and a sensitivity of 80.3 ± 19.2%, whilst acceleration-derived estimations had a specificity of 94.5 ± 6.4% and a sensitivity of 73.2 ± 15.8%. This study demonstrated that orthosis compliance can be monitored outside the laboratory, and, furthermore, this device offers insights into the intensity and frequency of a user’s activities and has the future potential to monitor orthosis fit and forces applied to affected joints using pressure.

## 1. Introduction

Orthoses are externally worn medical devices designed to support a joint, whether by stabilising or immobilising, or assisting with function or motion [1,2,3,4]. The use of orthoses is less invasive and lower in cost than alternative treatment options, such as surgery.

Defined as “the extent to which the patient’s behaviour matches the prescriber’s advice” [5], compliance to treatment plays a vital role in determining a treatment’s effectiveness. Levels of compliance towards orthosis treatment provides a deeper insight into patients’ behaviour towards orthoses use and ultimately aids clinicians to govern the course of the patient’s treatment [6]. Furthermore, monitoring compliance to orthotic treatment also provides valuable information in research settings looking into the effectiveness of both existing orthoses and novel orthotic interventions.

Recommended to relieve symptoms for a range of neurological and musculoskeletal conditions [7], orthoses have been designed to treat various anatomical regions, with design features dependent on the target condition of the orthosis. Yet, for various reasons, such as discomfort arising from skin irritation or sleep disturbance [8,9,10], patients stop wearing their orthoses as prescribed. With the UK’s annual expenditure on orthotic products and services amounting to at least £48 million [11] and with a growing number of users [12], when determining the effectiveness of orthotic treatment and to ensure the orthosis works as intended, it is essential to know whether patients wear their orthoses as prescribed [13].

When investigating the effectiveness of orthotic treatment, patient compliance is usually self-reported using patient diaries. However, this subjective approach often leads to patients overestimating their levels of compliance [8,14]. Approaches to quantifying compliance have been explored extensively in spinal orthoses [15,16,17,18,19,20]. Yet, in comparison, there is a lack of evidence documenting objective compliance monitoring for upper and lower limb orthoses [6]. Loss of function in the upper limb could occur at the fingers, hand, wrist, elbow, and shoulder, and thus, orthoses for the upper limb can come in various forms [21,22]. Additionally, many existing monitoring systems are either lab-based and not portable or require a direct connection to a computer to visualise or obtain monitoring data. Thus, the aim of this study was to validate the home use of an objective hand orthosis compliance monitoring device that was developed to be compact and obtain compliance data from a pressure sensor and an accelerometer wirelessly.

## 2. Materials and Methods

### 2.1. The Device

**Figure 1:**
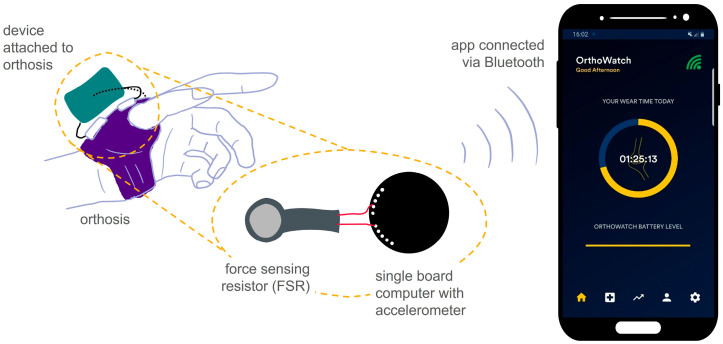
Components of the orthosis compliance monitoring device.

A wearable orthosis compliance monitoring device was designed comprising a force sensing resistor (FSR) attached to a single-board computer (MetaMotionC (MMC), Mbientlab Inc., San Francisco, CA, USA), with an in-built three-axis accelerometer that was connected via Bluetooth to a mobile phone application (Figure 1) [23]. Minimalistic and compact, the device was designed with a small form factor to be used alongside different orthosis types for various anatomical regions. The mobile phone application saved readings from the sensors to discern orthosis donning and doffing. The application was designed to show orthosis wear time and live battery level updates. It was programmed to save pressure and acceleration data from sensors at a rate of 1 Hz to a file on the phone whilst creating a new file every two hours to avoid data loss.

### 2.2. Experimental Protocol

Ethical approval for this study was obtained from the Imperial College Research Ethics Committee (ICREC reference: 21IC7308). Participants, aged between 18 and 80 years, were included in the study provided they had no history of upper limb pain or injury that compromised their ability to perform activities of daily living (ADLs) within the last 12 months.

Participants were given a device pack to take home and use for a week, which included: a Push^®^ ortho Thumb Brace CMC (Nea Company, Maastricht, The Netherlands) instrumented with the device, a phone with the device’s app installed, participant diary to log wear time, phone charging cable, two spare batteries, a screwdriver to aid with battery replacement, and two spare screws. During the week, participants were asked to wear the device for at least two hours a day. Additionally, participants were instructed to complete activities from a checklist whilst wearing the device. The checklist consisted of activities modified from the Arthritis Hand Function Test [24] and included the following tasks: preparing a meal, eating a meal with cutlery, pouring water or a drink, getting dressed, tying shoelaces, turning over pages in a book, texting on a phone, using a key to open a door, going on a walk, and one night’s sleep or a daytime nap. Participants were asked to tick off the activities they completed during the week and give a brief description of the activities they completed during the two hours they wore the device on their diary. At the end of the week, participants were required to complete the System Usability Scale (SUS) [25] to rate their experience with the device.

### 2.3. Data Analysis

The collected data underwent a filtering process consisting of, at every data point, a look up of sensor values from the preceding and subsequent 30 s. If there were any points in this time window where values were above the sensor’s threshold, the data point was assigned as one, translating to the orthosis being donned. For all other points in time, values were set to zero. To minimise sensor-to-sensor variability between the FSRs found in each device, an analogue-to-digital converter value of 10 counts was used as the threshold value for FSR data. Prior laboratory testing revealed that the specificity of accelerometer-based estimations required improvement [23], and, thus, to achieve a specificity close to 90%, optimal to estimate non-wear accurately [26], the acceleration threshold value was set to 1.061 g. The threshold was determined by averaging the vector magnitude of the acceleration obtained whilst participants completed the ADLs of the Southampton Hand Assessment Procedure (SHAP) [27] in laboratory testing of the device [23].

Percentage agreement between actual wear time and wear time estimated by each sensor was calculated as a measure of the device’s accuracy. Sensitivity, specificity, positive predictive value, negative predictive value, and the Cohen’s kappa coefficient were calculated for each sensor-based result. Differences between actual and sensor-derived wear times were checked for normal distribution using the Shapiro–Wilk test, after which paired t-tests (*p_shapiro-wilk_* > 0.05) or Wilcoxon signed-rank tests (*p_shapiro-wilk_* < 0.05) were used to check for any statistical differences.

To determine whether sampling rate affected device accuracy, sensor-based data were downsampled to emulate data being collected every 2, 5, 10, 15, 20, 30, and 60 s. For each sampling rate simulated, the accuracy of the wear time estimated was compared to that obtained originally at a sampling rate of 1 Hz using paired t-tests.

During the completion of the ADLs, device accuracy and acceleration vector magnitude were calculated. The acceleration vector magnitude was then resampled for each activity to normalise the time taken to complete the activity to a scale of 0–100%. To further explore the variation of activity levels between everyday tasks, the root mean square (RMS) of the acceleration during selected ADLs that represent essential daily activities of living vital for independent living was calculated. Namely, the RMSs of the acceleration during preparing a meal, eating with cutlery, getting dressed, walking, and sleeping were calculated and compared. Differences between paired comparisons were checked for normal distribution using the Shapiro–Wilk test, after which paired t-tests (*p_shapiro-wilk_* > 0.05) or Wilcoxon signed-rank tests (*p_shapiro-wilk_* < 0.05) were used to check for any statistical differences.

The usability of the compliance monitoring device, consisting of sensors connected to a mobile phone application, was calculated using the SUS. MATLAB (R2022b, The MathWorks, Inc., Natick, MA, USA), Microsoft Excel (v. 2409, Microsoft Corporation, Redmond, WA, USA), and SPSS Statistics for Windows (v. 26.0, IBM, Armonk, NY, USA) were used to carry out data analyses and visualisations.

## 3. Results

Ten healthy volunteers, aged between 23 and 54 years (mean: 29.7 ± 9.0 years; three males, seven females; Table S1), participated in the field testing of the device. The FSR and accelerometer-based compliance monitoring systems had overall accuracies of 96.9 ± 2.9% and 91.4 ± 4.9%, respectively. When the orthosis was donned, on average, pressure and acceleration data were collected for 85.0 ± 13.2% and 68.0 ± 8.3% of the time, respectively. Per participant, the device experienced Bluetooth disconnections 7.2 ± 4.2 times during the week. Only two participants needed to replace the device’s battery during the seven-day trial. Out of the ten participants, four participants wore their orthosis every day for seven days, as instructed, whilst two missed one day of orthosis wear and four missed two days.

After confirming differences were normally distributed, no differences were found between actual and estimated wear times, determined by both the FSR and the accelerometer (*p* = 0.171 and *p* = 0.451, respectively). Compliance data derived from the FSR had a specificity of 99.3 ± 0.7%, sensitivity of 80.3 ± 19.2%, positive predictive value of 94.1 ± 6.8%, and negative predictive value of 97.2 ± 3.4% (Table 1).

Wear time data obtained from the accelerometer had a specificity of 94.5 ± 6.4%, sensitivity of 73.2 ± 15.8%, positive predictive value of 70.1 ± 20.9%, and negative predictive value of 95.3 ± 4.1% (Table 2). The Cohen’s kappa coefficient had a mean of 0.83 ± 0.13 for the FSR’s wear time estimations and a mean of 0.63 ± 0.14 for the accelerometer.

Differences between the accuracy of wear time estimations obtained at the original sampling rate of 1 Hz and the downsampled wear time accuracies were normally distributed. All downsampled FSR-based wear time estimation accuracies differed from the accuracy at 1 Hz (*p* < 0.039). The accuracy of the accelerometer-determined wear time estimation at 1/60 Hz differed from that of the original sample rate (*p* = 0.027).

**Figure 2:**
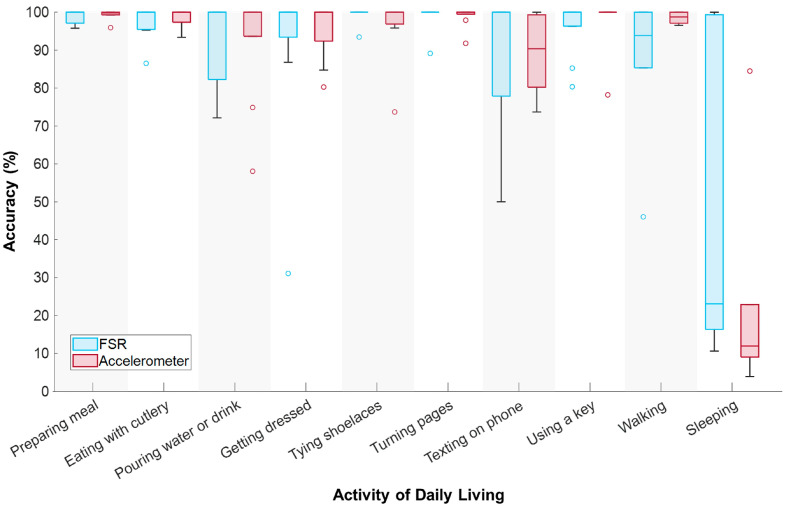
Accuracy of wear time estimations obtained using both FSR and accelerometer data whilst ten participants performed the activities of daily living they were instructed to complete when testing the device outside the laboratory.

The device’s accuracy of measuring compliance whilst participants performed ADLs revealed that wear time estimations had the lowest agreement with actual wear time when users slept (Figure 2).

Paired comparisons between the accuracies of pressure and acceleration-based compliance data during ADLs revealed that there were no differences between the two (*p* > 0.144).

**Figure 3:**
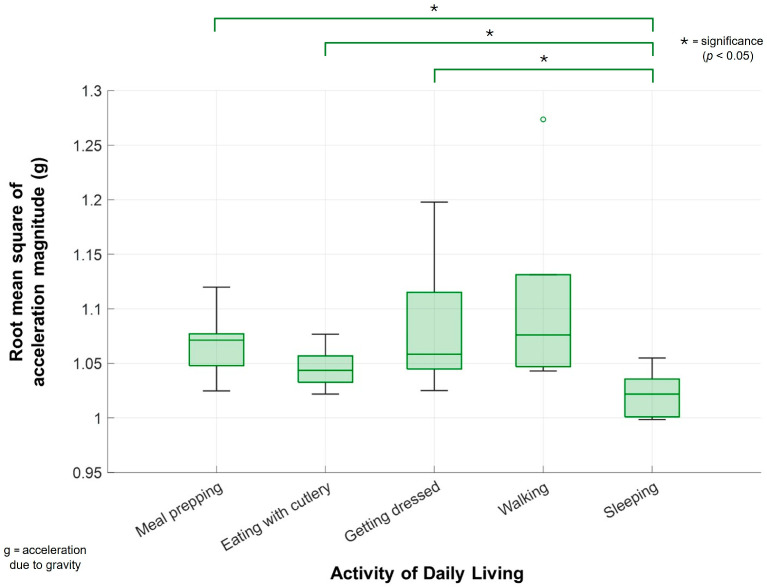
Boxplot of the root mean square values of the acceleration magnitude (where g = acceleration due to gravity) logged by ten participants during various activities of daily living.

**Figure 4:**
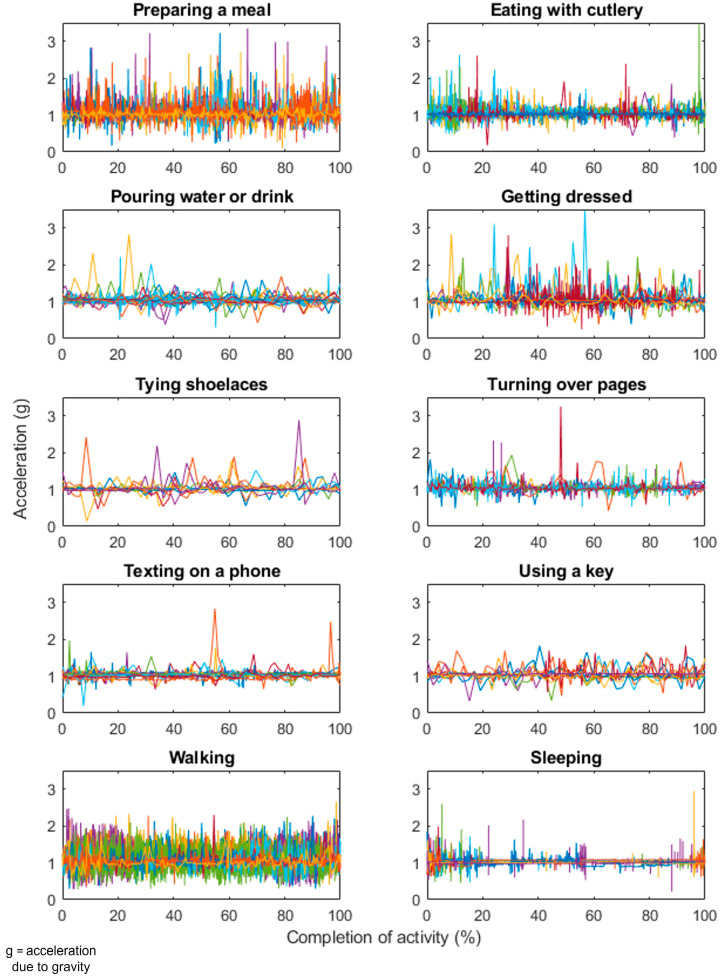
The vector magnitude of acceleration (g = acceleration due to gravity) captured by the three-axis accelerometer when ten participants completed activities of daily living. Each colour represents one trial, and participants could complete each ADL more than once.

Differences were found when comparing the RMS of the acceleration magnitudes recorded during sleeping and meal prepping (*p* = 0.003), eating (*p* = 0.010), and getting dressed (*p* = 0.046) (Figure 3). Changes in acceleration captured by the accelerometer revealed the range of participant activity levels when completing ADLs (Figure 4).

**Figure 5:**
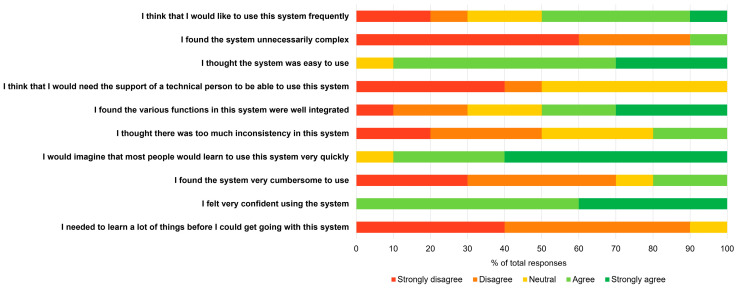
Mean scores for each item of the System Usability Scale (SUS) during the field testing of the compliance monitoring device.

An average SUS score of 73.8 ± 14.8 was obtained, and participants generally found the device simple and easy to use (Figure 5).

## 4. Discussion

There was no difference between the estimated wear times, from both the FSR and the accelerometer, when compared with actual wear time. However, the calculation of the Cohen’s kappa coefficient revealed that wear time determined using data from the FSR had a higher level of reliability (83%) than that from the accelerometer (63%). The Cohen’s kappa statistic classifies level of agreement as none (0–0.20), minimal (0.21–0.39), weak (0.40–0.59), moderate (0.60–0.79), strong (0.80–0.90), and almost perfect (0.90+) [28]. Thus, upon interpretation, there was a strong level of agreement between user-reported and FSR-based wear times and a moderate level of agreement between user-reported and accelerometer-based wear times. The implementation of a filter that considered values 30 s before and after the current time to determine wear resulted in more accurate wear time estimations than wear time estimates obtained by thresholding alone [23].

Calculations of the device’s accuracy during the completion of ADLs revealed wear time estimations had a lower percentage agreement with actual wear time during sleep than that during the other ADLs. One reason for this could be the lack of hand activity during this ADL, leading to acceleration values that were lower than the threshold value. Further, as the hand relaxes during sleep, there may be a decreased potential for forces at the orthosis–skin interface, especially if orthosis fit is not customised.

Visualising accelerometer data and calculations of the RMS of acceleration during the various ADLs revealed that the range of acceleration depended on the nature of the activity. ADLs that required minimal hand movement to complete, such as sleeping or texting on a phone, and ADLs that generally required slow and steady hand motion, such as pouring water or turning over a page, exhibited a smaller range of acceleration values than those for ADLs that required unrestricted, quick and precise hand movements, such as walking, preparing a meal, using cutlery, or getting dressed, and ADLs that required rotation of the hands to complete, such as tying shoelaces or using a key. Specifically, for ADLs such as sleeping, it is evident that, with acceleration values close to 1 g, there is minimal hand movement detected during the activity; the detected acceleration is all due to gravity. As previously mentioned, the accelerometer-based wear time estimations lacked sensitivity, but one way to better estimate wear in the future could be to use the existing dataset to preassign threshold values for common ADLs and then use these values to classify wear time by identifying the type of activity the user completes whilst wearing their orthosis. A similar method was implemented in a study investigating wear time of a lower limb orthoses; standard deviations of accelerometer values identified whilst sitting, standing, walking, and ascending and descending stairs were used to detect orthosis wear [29]. The variability of acceleration has also been used to identify shoulder sling wear time [26].

Comparisons between percentage agreement at the ranges of sampling rates demonstrated that, whilst a higher sampling rate had higher accuracy and, therefore, could be used to justify lowering the sampling rate to 1/30 Hz for the accelerometer, as there was no statistical difference in accuracy, it should be noted that data sampling rate could be affected by various aspects of the device, such as Bluetooth connection, memory, and timer settings, that may have not been represented when downsampling data to a particular sampling rate as opposed to collecting data at said rate.

One limitation of the device’s field testing is that it was not completed by current orthosis users. Whilst users wore the device for the recommended amount of time every day for a week, these users were aware of the aim of the study and were healthy volunteers. In contrast, those who are prescribed hand orthoses, are likely to have compromised hand function alongside condition-related symptoms that could affect the way they wear their orthosis, and an ideal field-testing study should include patient participation.

Additionally, whilst previous studies investigating orthosis compliance on healthy participants have had similar or lower sample sizes [29,30,31,32,33,34], there is no doubt that a higher sample size would contribute to eliminating the effects of data loss occurring from Bluetooth disconnections and users forgetting to complete their required daily wear.

Another limitation in this one-week trial is that actual wear time estimations relied on the time as recorded by users when they donned and doffed the device, which could be prone to recall bias if times were not recorded immediately. Thus, the study relied on user-reported wear time as the ground truth. However, an advantage of recruiting healthy participants is that they, when compared with patients, would not be affected by social desirability bias when self-reporting wear time as they were not prescribed their orthosis.

It should be noted that users faced compromised dexterity whilst wearing the orthosis during previous laboratory testing [23]. This could have caused individuals to adapt the way they would normally carry out ADLs, such as using cutlery. Thus, this could mean the data obtained, especially regarding activity from the accelerometer, may not be truly representative of users performing ADLs.

Device disconnections lead to loss of orthosis wear time data. Specifically, as the recommended wear time for each user was two hours a day, if there were any disconnections during this time, it would inevitably reduce the amount of wear time data collected. However, Bluetooth disconnections would make users reconnect the device themselves, especially if the loss of connection is noticed during daily wear time. Consequently, users who experienced disconnections during wear can actively reconnect back, but disconnections happening outside of daily wear time could be overlooked as users are away from their device, which could result in hours of non-wear data being lost.

Lastly, whilst volunteers were provided with mobile phones installed with the app, this experience cannot be assumed as equivalent to having an app downloaded to one’s own mobile phone. Thus, it may be possible that some Bluetooth disconnections that occurred during the week were due to users forgetting the extra mobile phone device given to them as part of the study, as no disconnections were reported whilst testing the device in the laboratory [23].

## 5. Conclusions

This study validated the use of monitoring hand orthosis compliance outside the laboratory, in the real world where such a device would be used. Monitoring orthosis wear time at home using a pressure sensor and accelerometer simultaneously has not been documented previously, and this device can benefit both clinical practice as well as research. Whilst the device can be used as it is to monitor wear time alone, the functionalities of the sensors included provide further insight into orthosis use and patient activity. Namely, the FSR could be used to explore forces applied to affected joints and orthosis fit. Similarly, the accelerometer also provides information regarding the user’s movement whilst they wear their orthosis. Ultimately, given its minimalistic form factor, this device should be able to work alongside any type of orthosis, giving orthotic users a way to track their usage and wear time. Equally important, the device can also be utilised in research studies quantifying compliance to orthosis recommendations or in studies examining the efficacy of both existing and novel orthoses.

## Figures and Tables

**Table 1:** Sensitivity, specificity, positive predictive value, negative predictive value, and Cohen’s kappa of pressure-derived wear time estimations.

Parameter	Mean ± Standard Deviation	95% Confidence Intervals
Sensitivity	80.3 ± 19.2	66.5–94.0
Specificity	99.3 ± 0.7	98.9–99.8
Positive predictive value	94.1 ± 6.8	89.2–99.0
Negative predictive value	97.2 ± 3.4	94.7–99.6
Cohen’s kappa	0.83 ± 0.13	0.74–0.93

**Table 2:** Sensitivity, specificity, positive predictive value, negative predictive value, and Cohen’s kappa of acceleration-derived wear time estimations.

Parameter	Mean ± Standard Deviation	95% Confidence Intervals
Sensitivity	73.2 ± 15.8	61.9–84.6
Specificity	94.5 ± 6.4	89.9–99.1
Positive predictive value	70.1 ± 20.9	55.1–85.0
Negative predictive value	95.3 ± 4.1	92.4–98.3
Cohen’s kappa	0.63 ± 0.14	0.54–0.73

## Data Availability

The original contributions presented in this study are included in the article/Supplementary Material. Further inquiries can be directed to the corresponding author.

## Supplementary Materials

The following supporting information can be downloaded at https://www.mdpi.com/article/10.3390/bioengineering13060697/s1: Table S1: Participant demographics. Table S2: Device accuracy (Force Sensing Resistor; FSR). Table S3: Percentage agreement vs. sampling rates (FSR). Table S4: Percentage agreement during activities of daily living (FSR). Table S5: Device accuracy (accelerometer). Table S6: Percentage agreement vs. sampling rates (accelerometer). Table S7: Percentage agreement during activities of daily living (accelerometer). Table S8: Results from System Usability Scale (SUS). Table S9: Paired groups and Shapiro–Wilk test for normality. Table S10: Paired *t*-test results. Table S11: Wilcoxon signed-rank test results.

## Abbreviations

The following abbreviations are used in this manuscript:

FSR	Force sensing resistor
ADL	Activities of daily living
RMS	Root mean square
SUS	System Usability Scale
