# Supplementary material for: Practicality of Using Pressure Sensors and Accelerometers to Quantify Hand Orthosis Compliance at Home
Source: Bioengineering (Basel). 2026 Jun 18;13(6):697. doi: 10.3390/bioengineering13060697 (PMC13296036; doi:10.3390/bioengineering13060697)
Supplement: Supplementary file 1 [file bioengineering-13-00697-s001.zip › bioengineering-4339875-supplementary.pdf]

SUPPLEMENTARY MATERIAL

TABLE S1: PARTICIPANT DEMOGRAPHICS

	1	2	3	4	5	6	7	8	9	10
Age (years)	32	26	27	24	23	30	28	28	54	25
Gender	F	M	F	F	F	M	F	F	F	M
Push Brace size	R1	L1	R1	R1	R2	R1	R1	R1	R2	R1

TABLE S2: DEVICE ACCURACY (FORCE SENSING RESISTOR; FSR)

	True Positive	False Positive	False Negative	True Negative	Sensitivity (%)	Specificity (%)	Positive Predictive Value (%)	Negative Predictive Value (%)	Percentage Agreement (%)	Cohen's Kappa
1	52470	9598	5707	535156	90.19	98.24	84.54	98.94	97.46	0.86
2	52568	1965	1157	404786	97.85	99.52	96.40	99.71	99.32	0.97
3	18752	487	6826	330818	73.31	99.85	97.47	97.98	97.95	0.83
4	35684	1345	40049	392318	47.12	99.66	96.37	90.74	91.18	0.59
5	22047	6052	0	315686	100.00	98.12	78.46	100.00	98.24	0.87
6	34698	662	932	485096	97.38	99.86	98.13	99.81	99.69	0.98
7	50384	544	0	57547	100.00	99.06	98.93	100.00	99.50	0.99
8	20097	787	8698	403019	69.79	99.81	96.23	97.89	97.81	0.80
9	47422	1427	22495	365440	67.83	99.61	97.08	94.20	94.52	0.77
10	35682	1024	24551	303990	59.24	99.66	97.21	92.53	93.00	0.70

TABLE S3: PERCENTAGE AGREEMENT VS. SAMPLING RATES (FSR)

	1 Hz	1/2 Hz	1/5 Hz	1/10 Hz	1/15 Hz	1/20 Hz	1/30 Hz	1/60 Hz
1	97.46	97.28	96.90	96.57	96.41	96.35	95.99	94.63
2	99.32	99.29	99.24	99.16	99.10	99.04	98.87	97.93
3	97.95	97.83	97.58	97.34	97.20	97.22	96.90	95.95
4	91.18	90.45	89.21	88.44	87.88	88.15	87.26	85.68
5	98.24	98.27	98.26	98.30	98.29	98.26	98.34	98.45
6	99.69	99.66	99.62	99.60	99.59	99.59	99.53	99.11
7	99.50	99.49	99.48	99.47	99.47	99.45	99.42	98.95
8	97.81	97.67	97.53	97.35	97.27	97.25	97.20	96.59
9	94.52	94.34	94.14	93.83	93.52	93.53	93.16	91.15
10	93.00	92.38	91.27	90.28	89.80	89.76	88.77	86.41

TABLE S4: PERCENTAGE AGREEMENT DURING ACTIVITIES OF DAILY LIVING (FSR)

	Preparing meal	Eating with cutlery	Pouring water or drink	Getting dressed	Tying shoelaces	Turning pages	Texting on phone	Using a key	Walking	Sleeping
1	100.00	100.00	100.00	86.78	100.00	100.00	77.85	100.00	46.03	100.00
2	100.00	100.00	72.13	100.00	100.00	100.00	100.00	80.33	100.00	99.33
3	100.00	100.00	-	100.00	-	100.00	100.00	100.00	-	26.96
4	100.00	95.49	84.70	100.00	93.44	100.00	60.58	85.25	87.68	10.63
5	-	100.00	100.00	-	100.00	100.00	100.00	100.00	-	-
6	-	-	100.00	-	-	100.00	100.00	100.00	100.00	-
7	100.00	100.00	100.00	100.00	100.00	100.00	100.00	100.00	-	-
8	-	100.00	74.90	31.08	-	-	50.00	-	-	-
9	95.76	95.27	99.92	100.00	100.00	89.14	97.30	100.00	85.31	19.23
10	96.12	86.51	100.00	100.00	100.00	100.00	100.00	100.00	100.00	16.34

TABLE S5: DEVICE ACCURACY (ACCELEROMETER)

	True Positive	False Positive	False Negative	True Negative	Sensitivity (%)	Specificity (%)	Positive Predictive Value (%)	Negative Predictive Value (%)	Percentage Agreement (%)	Cohen's Kappa
1	37493	9202	7951	415746	82.50	97.83	80.29	98.12	96.36	0.79
2	29556	22311	12215	293344	70.76	92.93	56.98	96.00	90.36	0.58
3	13622	6592	6312	251168	68.34	97.44	67.39	97.55	95.36	0.65
4	32264	4362	26533	300931	54.87	98.57	88.09	91.90	91.51	0.63
5	17076	9463	4296	237785	79.90	96.17	64.34	98.23	94.88	0.69
6	26968	64614	836	313198	96.99	82.90	29.45	99.73	83.86	0.39
7	33081	436	6323	44986	83.95	99.04	98.70	87.68	92.03	0.84
8	14663	2375	7783	311182	65.33	99.24	86.06	97.56	96.98	0.73
9	46701	48898	7704	236423	85.84	82.86	48.85	96.84	83.34	0.53
10	20700	4996	26455	233475	43.90	97.90	80.56	89.82	88.99	0.51

TABLE S6: PERCENTAGE AGREEMENT VS. SAMPLING RATES (ACCELEROMETER)

	1 Hz	1/2 Hz	1/5 Hz	1/10 Hz	1/15 Hz	1/20 Hz	1/30 Hz	1/60 Hz
1	96.36	95.65	94.31	93.26	92.55	92.88	92.11	91.42
2	90.36	89.85	88.85	88.57	88.28	88.56	88.28	88.41
3	95.36	95.51	95.11	94.80	93.59	94.68	94.19	93.53
4	91.51	90.36	88.62	87.27	86.64	86.62	85.98	84.90
5	94.88	94.49	93.51	92.85	92.72	92.87	92.07	92.03
6	83.86	86.27	89.44	90.90	91.12	91.43	91.79	65.74
7	92.03	87.07	78.78	72.40	70.25	68.94	65.56	60.16
8	96.98	96.53	95.64	95.10	94.88	94.81	94.56	93.95
9	83.34	83.27	82.92	82.39	81.78	81.95	80.75	79.09
10	88.99	88.41	86.96	86.03	85.93	85.65	85.12	84.11

TABLE S7: PERCENTAGE AGREEMENT DURING ACTIVITIES OF DAILY LIVING (ACCELEROMETER)

	Preparing meal	Eating with cutlery	Pouring water or drink	Getting dressed	Tying shoelaces	Turning pages	Texting on phone	Using a key	Walking	Sleeping
1	99.56	100.00	100.00	84.74	100.00	100.00	83.83	100.00	99.21	22.89
2	100.00	100.00	100.00	100.00	100.00	100.00	100.00	100.00	98.25	9.67
3	100.00	98.37	-	100.00	-	100.00	96.90	100.00	-	14.25
4	99.79	93.37	58.04	100.00	73.68	100.00	100.00	100.00	96.50	9.05
5	-	99.49	100.00	-	95.81	100.00	75.00	100.00	-	-
6	-	-	100.00	-	-	100.00	99.23	78.19	100.00	-
7	95.91	100.00	100.00	100.00	100.00	97.88	99.30	100.00	-	-
8	-	100.00	74.87	80.26	-	-	73.68	-	-	-
9	99.20	100.00	99.89	100.00	100.00	91.79	80.21	100.00	97.08	84.47
10	100.00	94.26	100.00	100.00	100.00	100.00	80.56	100.00	100.00	3.91

TABLE S8: RESULTS FROM SYSTEM USABILITY SCALE (SUS)

	1	2	3	4	5	6	7	8	9	10
I think that I would like to use this system frequently	1	4	2	4	3	3	1	4	4	5
I found the system unnecessarily complex	2	1	1	1	1	4	2	1	1	2
I thought the system was easy to use	4	4	4	5	4	4	4	5	3	5
I think that I would need the support of a technical person to be able to use this system	1	2	1	1	1	3	3	3	3	3
I found the various functions in this system were well integrated	2	4	3	5	3	2	1	4	5	5
I thought there was too much inconsistency in this system	4	4	3	1	2	2	3	2	3	1
I would imagine that most people would learn to use this system very quickly	5	5	3	5	5	4	4	4	5	5
I found the system very cumbersome to use	4	3	2	1	2	2	4	1	1	2
I felt very confident using the system	4	4	4	5	4	4	4	5	5	5
I needed to learn a lot of things before I could get going with this system	2	1	1	1	2	2	2	2	3	1

TABLE S9: PAIRED GROUPS & SHAPIRO-WILK TEST FOR NORMALITY

	Paired groups		Shapiro – Wilk results		
#	Group 1	Group 2	Statistic	df	Sig.
1	Actual wear time	FSR-estimated wear time	0.784	5	0.060
2	Actual wear time	Accelerometer-estimated wear time	0.835	5	0.151
3	FSR accuracy at original sampling rate (1 Hz)	FSR accuracy at sampling rate of 1/2 Hz	0.790	5	0.067
4	FSR accuracy at original sampling rate (1 Hz)	FSR accuracy at sampling rate of 1/5 Hz	0.803	5	0.086
5	FSR accuracy at original sampling rate (1 Hz)	FSR accuracy at sampling rate of 1/10 Hz	0.841	5	0.168
6	FSR accuracy at original sampling rate (1 Hz)	FSR accuracy at sampling rate of 1/15 Hz	0.835	5	0.153
7	FSR accuracy at original sampling rate (1 Hz)	FSR accuracy at sampling rate of 1/20 Hz	0.862	5	0.234
8	FSR accuracy at original sampling rate (1 Hz)	FSR accuracy at sampling rate of 1/30 Hz	0.887	5	0.340
9	FSR accuracy at original sampling rate (1 Hz)	FSR accuracy at sampling rate of 1/60 Hz	0.968	5	0.859
10	Accelerometer accuracy at original sampling rate (1 Hz)	Accelerometer accuracy at sampling rate of 1/2 Hz	0.987	5	0.969
11	Accelerometer accuracy at original sampling rate (1 Hz)	Accelerometer accuracy at sampling rate of 1/5 Hz	0.982	5	0.946
12	Accelerometer accuracy at original sampling rate (1 Hz)	Accelerometer accuracy at sampling rate of 1/10 Hz	0.983	5	0.952
13	Accelerometer accuracy at original sampling rate (1 Hz)	Accelerometer accuracy at sampling rate of 1/15 Hz	0.853	5	0.203
14	Accelerometer accuracy at original sampling rate (1 Hz)	Accelerometer accuracy at sampling rate of 1/20 Hz	0.960	5	0.809
15	Accelerometer accuracy at original sampling rate (1 Hz)	Accelerometer accuracy at sampling rate of 1/30 Hz	0.971	5	0.882
16	Accelerometer accuracy at original sampling rate (1 Hz)	Accelerometer accuracy at sampling rate of 1/60 Hz	0.880	5	0.309
17	FSR accuracy – preparing a meal	Accelerometer accuracy – preparing a meal	0.640	5	0.002*
18	FSR accuracy – eating with cutlery	Accelerometer accuracy – eating with cutlery	0.859	5	0.226
19	FSR accuracy – pouring water/drink	Accelerometer accuracy – pouring water/drink	0.883	5	0.324
20	FSR accuracy – getting dressed	Accelerometer accuracy – getting dressed	0.552	5	0.000*
21	FSR accuracy – tying shoelaces	Accelerometer accuracy – tying shoelaces	0.682	5	0.006*
22	FSR accuracy – turning pages	Accelerometer accuracy – turning pages	0.686	9	0.001*
23	FSR accuracy – texting on phone	Accelerometer accuracy – texting on phone	0.936	5	0.637
24	FSR accuracy – using a key	Accelerometer accuracy – using a key	0.753	5	0.032*
25	FSR accuracy – walking	Accelerometer accuracy – walking	0.770	5	0.045*
26	FSR accuracy – sleeping	Accelerometer accuracy – sleeping	0.934	5	0.624
27	RMS of acceleration – meal prepping	RMS of acceleration – eating with cutlery	0.973	5	0.895
28	RMS of acceleration – meal prepping	RMS of acceleration – getting dressed	0.802	5	0.084
29	RMS of acceleration – meal prepping	RMS of acceleration – walking	0.834	5	0.148
30	RMS of acceleration – meal prepping	RMS of acceleration – sleeping	0.907	5	0.450
31	RMS of acceleration – eating with cutlery	RMS of acceleration – getting dressed	0.974	5	0.900
32	RMS of acceleration – eating with cutlery	RMS of acceleration – walking	0.882	5	0.316
33	RMS of acceleration – eating with cutlery	RMS of acceleration – sleeping	0.912	5	0.483
34	RMS of acceleration – getting dressed	RMS of acceleration – walking	0.740	5	0.024*
35	RMS of acceleration – getting dressed	RMS of acceleration – sleeping	0.961	5	0.817
36	RMS of acceleration – walking	RMS of acceleration – sleeping	0.914	5	0.495

(* $p < 0.05$ = statistically different)

TABLE S10: PAIRED T-TEST RESULTS

#	Paired groups		Paired t-test results					
	Group 1	Group 2	Mean	Standard deviation	t-statistic	df	p-value	Cohen's d
1	Actual wear time	FSR-estimated wear time	-7457.00	15836.25	-1.489	9	0.171	-0.471
2	Actual wear time	Accelerometer-estimated wear time	6684.10	26799.72	0.789	9	0.451	0.249
3	FSR accuracy at original sampling rate - 1 Hz	FSR accuracy at sampling rate of 1/2 Hz	0.20	0.26	2.464	9	0.036*	0.779
4	FSR accuracy at original sampling rate - 1 Hz	FSR accuracy at sampling rate of 1/5 Hz	0.54	0.71	2.409	9	0.039*	0.762
5	FSR accuracy at original sampling rate - 1 Hz	FSR accuracy at sampling rate of 1/10 Hz	0.83	1.05	2.522	9	0.033*	0.797
6	FSR accuracy at original sampling rate - 1 Hz	FSR accuracy at sampling rate of 1/15 Hz	1.01	1.24	2.585	9	0.029*	0.817
7	FSR accuracy at original sampling rate - 1 Hz	FSR accuracy at sampling rate of 1/20 Hz	1.01	1.18	2.697	9	0.025*	0.853
8	FSR accuracy at original sampling rate - 1 Hz	FSR accuracy at sampling rate of 1/30 Hz	1.33	1.54	2.713	9	0.024*	0.858
9	FSR accuracy at original sampling rate - 1 Hz	FSR accuracy at sampling rate of 1/60 Hz	2.38	2.22	3.391	9	0.008*	1.072
10	Accelerometer accuracy at original sampling rate - 1 Hz	Accelerometer accuracy at sampling rate of 1/2 Hz	0.63	1.80	1.097	9	0.301	0.347
11	Accelerometer accuracy at original sampling rate - 1 Hz	Accelerometer accuracy at sampling rate of 1/5 Hz	1.95	4.61	1.338	9	0.214	0.423
12	Accelerometer accuracy at original sampling rate - 1 Hz	Accelerometer accuracy at sampling rate of 1/10 Hz	3.01	6.61	1.441	9	0.183	0.456
13	Accelerometer accuracy at original sampling rate - 1 Hz	Accelerometer accuracy at sampling rate of 1/15 Hz	3.59	7.18	1.582	9	0.148	0.500
14	Accelerometer accuracy at original sampling rate - 1 Hz	Accelerometer accuracy at sampling rate of 1/20 Hz	3.53	7.65	1.457	9	0.179	0.461
15	Accelerometer accuracy at original sampling rate - 1 Hz	Accelerometer accuracy at sampling rate of 1/30 Hz	4.33	8.60	1.590	9	0.146	0.503
16	Accelerometer accuracy at original sampling rate - 1 Hz	Accelerometer accuracy at sampling rate of 1/60 Hz	8.03	9.62	2.639	9	0.027*	0.835
18	FSR accuracy – eating with cutlery	Accelerometer accuracy – eating with cutlery	-0.912	3.207	-0.853	8	0.418	-0.284
19	FSR accuracy – pouring water/drink	Accelerometer accuracy – pouring water/drink	-0.129	13.634	-0.028	8	0.978	-0.009
23	FSR accuracy – texting on phone	Accelerometer accuracy – texting on phone	-0.297	19.595	-0.048	9	0.963	-0.015
26	FSR accuracy – sleeping	Accelerometer accuracy – sleeping	21.373	56.249	0.931	5	0.395	0.380
27	RMS of acceleration – meal prepping	RMS of acceleration – eating with cutlery	0.021	0.031	2.092	8	0.070	0.697
28	RMS of acceleration – meal prepping	RMS of acceleration – getting dressed	-0.023	0.058	-1.172	8	0.275	-0.391
29	RMS of acceleration – meal prepping	RMS of acceleration – walking	-0.038	0.084	-1.101	5	0.321	-0.449
30	RMS of acceleration – meal prepping	RMS of acceleration – sleeping	0.049	0.023	5.197	5	0.003*	2.122
31	RMS of acceleration – eating with cutlery	RMS of acceleration – getting dressed	-0.028	0.053	-1.496	7	0.178	-0.529
32	RMS of acceleration – eating with cutlery	RMS of acceleration – walking	-0.062	0.101	-1.520	5	0.189	-0.621
33	RMS of acceleration – eating with cutlery	RMS of acceleration – sleeping	0.020	0.012	3.990	5	0.010*	1.629
35	RMS of acceleration – getting dressed	RMS of acceleration – sleeping	0.060	0.056	2.634	5	0.046*	1.075
36	RMS of acceleration – walking	RMS of acceleration – sleeping	0.092	0.098	2.082	4	0.106	0.931

(* $p < 0.05$ = statistically different)

TABLE S11: WILCOXON SIGNED-RANK TEST RESULTS

	Paired groups		Wilcoxon signed-rank test results	
#	Group 1	Group 2	Z-value	p-value
17	FSR accuracy – preparing a meal	Accelerometer accuracy – preparing a meal	-0.135	0.893
20	FSR accuracy – getting dressed	Accelerometer accuracy – getting dressed	-0.447	0.655
21	FSR accuracy – tying shoelaces	Accelerometer accuracy – tying shoelaces	-1.342	0.180
22	FSR accuracy – turning pages	Accelerometer accuracy – turning pages	-0.447	0.655
24	FSR accuracy – using a key	Accelerometer accuracy – using a key	0.000	1.000
25	FSR accuracy – walking	Accelerometer accuracy – walking	-1.461	0.144
34	RMS of acceleration – getting dressed	RMS of acceleration – walking	-0.135	0.893
